# Chronic aseptic meningitis caused by enterovirus in a humorally immunosuppressed adult patient presenting with sensorineural hearing loss: a case report

**DOI:** 10.1186/s12879-021-06956-1

**Published:** 2022-01-04

**Authors:** Sean M. Anderson, Daniel Gold, Gregory Olson, Jennifer Pisano

**Affiliations:** 1 Division of Internal Medicine, The University of Chicago Medicine, 5841 S Maryland Avenue, Suite 3C, Chicago, IL 60637 USA; 2 Division of Infectious Diseases, The University of Chicago Medicine, 5841 S Maryland Avenue, Suite 5D, Chicago, IL 60637 USA

**Keywords:** Enterovirus, Aseptic meningitis, Rituximab

## Abstract

**Background:**

Enterovirus has been described as a cause of aseptic meningitis in humorally immunosuppressed patients.

**Case presentation:**

A 67-year-old female with a history of mantle cell lymphoma on rituximab therapy presented with subacute hepatitis, myalgias, and sensorineural hearing loss several months after an initial febrile illness. She was diagnosed with enterovirus infection by CSF PCR as a unifying etiology of her presentation, representing an unusual presentation of disease.

**Discussion and conclusions:**

This patient’s unique presentation and clinical course presents important implications in the care of similarly immunosuppressed patients with cryptic complaints.

## Background

Enteroviruses are RNA viruses within the *Picornaviridae* family that are ubiquitous and commonly passed between humans via the fecal–oral route. The viruses replicate initially in the pharynx and terminal ileum before giving rise to two phases of viremia. First, the “minor” phase of viremia occurs during which the viruses spread to the lymphoid tissues of the body for further replication. This ongoing replication contributes to the “major” phase of viremia, which is responsible for the majority of clinical symptoms as well as viral seeding of target tissues including the central nervous system (CNS). The incubation periods for the stages of viremia are not well-defined. The primary immune mechanism combating severe infection, especially re-infection with a serotype to which an individual has previously been exposed, is the humoral (B-cell) response, particularly secretory IgA within the gut [1]. While the vast majority of enteroviral infections in adults result in asymptomatic disease with a rapidly clearing viremia, there have been several documented cases of severe, CNS-predominant chronic enterovirus infections in patients with primary or iatrogenic hypogammaglobulinemia [1–5]. Rituximab is a monoclonal antibody against CD-20 that preferentially targets B-cells for antibody-dependent cellular toxicity. When used therapeutically for diseases including certain cancers and autoimmune conditions, rituximab depletes B-cell stores, and thus immunoglobulin production. There are several documented cases connecting rituximab therapy, specifically as a treatment for hematologic malignancy, with severe enteroviral infection [3, 4].

## Objective

To report the clinical course and diagnosis of aseptic meningitis, subacute hepatitis, and myositis caused by a unique presentation of disseminated enterovirus infection in a humorally immunosuppressed patient.

## Case presentation

A 67-year-old female actively practicing pediatrician with a history of mantle cell lymphoma on maintenance rituximab therapy and documented uncomplicated hypogammaglobulinemia presented to the hospital in October with three months of progressive myalgias, fatigue, and decreased appetite. She was initially treated with three cycles of R-CHOP and three cycles of R-DHAP for her blastoid variant mantle cell lymphoma followed by autologous stem cell transplantation with BEAM conditioning. She was D + 1045 in first complete remission and receiving rituximab infusions every two months at her time of presentation.

Her symptoms started with a 4-week illness in July characterized by low-grade fevers, myalgias, fatigue, and nausea. Although the fevers resolved as an outpatient, her other symptoms persisted, prompting an evaluation by her primary care physician in September, where lab work demonstrated normal renal function and electrolytes, an aspartate aminotransferase (AST) of 606 U/L (ref 8–37 U/L), alanine aminotransferase (ALT) of 698 U/L (ref 8–37 U/L), alkaline phosphatase (AP) of 631 U/L (ref 50–150 U/L), an albumin of 3.8 g/dL, and a total bilirubin of 1.4 mg/dL (ref 0.1–1.0). Further rituximab infusions were discontinued at this time. An outpatient workup for the etiology of her liver disease was performed. A positron emission tomography scan for recurrence of lymphoma was negative. Serum polymerase chain reaction testing (PCR) for EBV, CMV, HSV-1, HSV-2, adenovirus, and HHV-6 were negative. Serologic testing for hepatitis A was positive for IgG and negative for IgM. Hepatitis B surface antigen and core antibody, hepatitis C antibody, and hepatitis E antibody were negative. Ceruloplasmin was normal and alpha-1 antitrypsin, antimitochondrial antibody, and antinuclear antibody were negative. Magnetic resonance imaging (MRI) of the liver with and without contrast showed hepatomegaly, mild periportal edema, and mild prominence of the intrahepatic bile ducts. A transjugular liver biopsy revealed a mild lymphocytic infiltrate, no evidence of fibrosis or steatosis, and no evidence of lymphoma, and was interpreted as resolving acute hepatitis. The patient had an elevated myoglobin in the urine to 48 ug/L (ref < 20 ug/L), and an elevated serum creatine kinase of 263 U/L (ref 29–143 U/L).

Two weeks before presentation to a quaternary-care hospital in mid-October, she developed gradually progressive bilateral hearing loss and worsening myalgias without reported headache or neck stiffness. By the time of admission, her liver enzymes had improved with an AST of 262, ALT of 202, AP of 197, and a total bilirubin of 0.5. Serum albumin had fallen to 1.9 g/dL (ref 3.5–5.0 g/dL) and international normalized ratio (INR) was 1.0. Initial examination was most remarkable for diffuse pitting edema. Neurologic examination uncovered mild bilateral sensorineural hearing loss and 3 + /5 proximal upper extremity muscle strength without any other abnormalities. Her hospital course was complicated by the continued progression of her hearing loss. At first, she experienced difficulty hearing voices if words were spoken softly. One week later, she could hear virtually no noise and required a writing board for all communication. She was found to have a low-level detectable CMV PCR of 55.3 IU/mL in the peripheral blood, which was thought to be clinically insignificant viral reactivation in the setting of acute illness. Serum immunoglobulin studies showed a pan-hypogammaglobulinemia, including an IgG level of 241 mg/dL (ref 800–1700 mg/dL) from a baseline of 652 mg/dL as an outpatient. MRI of the brain and internal auditory canal with contrast were performed and demonstrated mild diffuse dural enhancement without any obvious retrocochlear or inner ear lesions or mass lesions. She was trialed on a 6-day course of prednisone 60 mg/day under a working diagnosis of vestibular neuritis with no effect. An audiogram demonstrated complete and partial sensorineural hearing loss on the right and left sides, respectively.

**Fig. 1:**
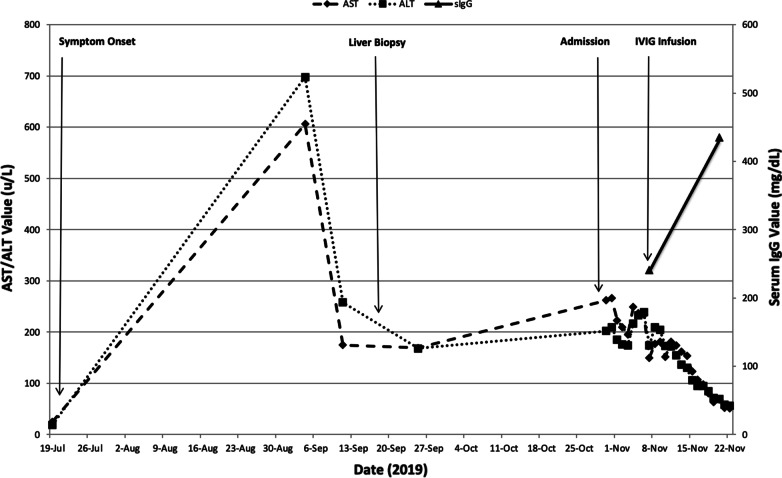
Key biochemical markers and events during patient's clinical course

A lumbar puncture was performed, and cerebrospinal fluid (CSF) analysis showed 21 white blood cells per uL (ref 0–5 WBC/uL) with 87% lymphocytes, 1 red blood cell per uL (ref 0–8 RBC/uL), CSF protein of 27 mg/dL (ref 15–45 mg/dL), and CSF glucose of 21 mg/dL (ref 50–70 mg/dL). A rapid CSF pathogen PCR (BioFire) panel was performed and resulted positive for enterovirus nucleic acid. CSF bacterial, fungal, and acid-fast bacilli cultures grew no organisms. CSF VDRL was negative. Shortly after this finding, she received infusions of intravenous immune globulin (IVIG) for therapy, first on hospital day 9 and then on hospital day 23 after a re-analysis of serum IgG levels showed improvement but persistent hypogammaglobulinemia (IgG level of 435 mg/dL). She was discharged to a subacute rehab facility on hospital day 24 with plans to recheck serum IgG levels every 2 weeks and administer IVIG if her level fell below 400 mg/dL. Figure 1 displays the trend of the patient’s AST and ALT over time as well as her recorded IgG levels. An improvement is noted in all markers following the initial dose of IVIG. As an outpatient, her serum IgG improved to 705 mg/dL following the two inpatient infusions of IVIG and she did not require a third infusion. Although her fatigue and myalgias improved, a repeat audiogram two months following her hospital discharge demonstrated little improvement in her auditory function. She is currently being worked up as a candidate for cochlear implant therapy.

## Discussion and conclusions

This patient’s long outpatient and inpatient route to a unifying diagnosis offers unique lessons in host–pathogen biology in the context of iatrogenic immunosuppression. Since the advent of immunotherapy as a novel paradigm in cancer treatment, there has been a perpetual tradeoff between therapeutic depletion of the immune system and the risk of infection. As mentioned in the introduction, humoral immunity has been described as a crucial mechanism of defense against enterovirus infection. This patient had known hypogammaglobulinemia as a consequence of her maintenance rituximab therapy, had a likely exposure to enterovirus working as a pediatrician during the summer, and developed progressive, multi-organ damage including debilitating CNS symptoms as a consequence of the infection. Despite this, enteroviral infection was not strongly considered as a unifying diagnosis until she developed bilateral hearing loss and had CSF analysis performed. Enterovirus causing deafness has been reported in the literature, albeit far from extensively [6, 7]. The use of IVIG infusions as a strategy to treat severe enterovirus infection in immunocompromised patients lacks robust clinical evidence due to a lack of clinical trials and is limited to case reports [8, 9]. However, it is an option for refractory or disease in these patients, and therefore a higher clinical suspicion for this disease in the setting of the above clinical indicators may have facilitated earlier and/or more aggressive treatment and improved the clinical outcome.

While chronic systemic enteroviral infection represents the most likely cause of this patient’s symptoms, we must mention the possibility of acute hepatitis virus infection despite the negativity of serologic testing (which can become falsely negative in the setting of Rituximab use) given that cases of similar clinical presentations have been reported to be caused by hepatitis B and C [10]. Additionally, drug-induced liver injury (e.g. from Rituximab) and occult heavy-metal toxicity are unable to be fully ruled out given the non-specific imaging and pathology findings. Finally, it must be noted that the patient did not have a documented IgG level for more than 2 years prior to presentation, although she did qualify as hypogammaglobulinemic at that time.

We advise physicians managing humorally immunosuppressed patients to consider a diagnosis of enterovirus infection under the correct clinical context, including the development of cryptic neurologic, hepatic, and/or musculoskeletal disease, and to pursue effective and timely treatment.

## Data Availability

The datasets used and/or analysed during the current study are available from the corresponding author on reasonable request.

## Abbreviations

CNS: Central nervous system
R-CHOP: Rituximab, cyclophosphamide, doxorubicin, vincristine, prednisone
R-DHAP: Rituximab, dexamethasone, cytarabine, platinol
BEAM: Carmustine, etoposide, cytarabine, melphelan
AST: Aspartate aminotransferase
ALT: Alanine aminotransferase
AP: Alkaline phosphatase
U/L: Units per liter
Ref: Reference range
INR: International normalized ratio
EBV: Epstein–Barr virus
HSV-1: Herpes simplex virus type 1
HSV-2: Herpes simplex virus type 2
HHV-6: Human Herpes virus type 6
CMV: Cytomegalovirus
PCR: Polymerase chain reaction
IgG: Immunoglobulin G
IgM: Immunoglobulin M
MRI: Magnetic resonance imaging
IVIG: Intravenous immune globulin
CSF: Cerebrospinal fluid
VDRL: Venereal disease research laboratory test

## References

[1] McKinney RE, Katz SL, Wilfert CM (1987). Chronic enteroviral meningoencephalitis in agammaglobulinemic patients. Rev Infect Dis.

[2] Quartier P, Tournilhac O, Archimbaud C (2003). Enteroviral meningoencephalitis after anti CD20 (Rituximab) treatment. Clin Infect Dis.

[3] Grisariu S, Vaxman I, Gatt M (2016). Enteroviral infection in patients treated with rituximab for non-Hodgkin lymphoma: a case series and review of the literature. Hematol Oncol.

[4] Servais S, Caers J, Warling O (2010). Enteroviral meningoencephalitis as complication of rituximab therapy in a patient treated for diffuse large B-cell lymphoma. Br J Haematol.

[5] Tellez R, Lastinger AM, Hogg JP (2019). Chronic enteroviral meningoencephalitis in a patient on rituximab for the treatment of psoriatic arthritis: a case report and brief literature review. IDCases.

[6] Schattner A (2003). Enteroviruses and sudden deafness. CMAJ.

[7] Mentel R, Kaftan H, Wegner U, Reissmann A, Gürtler L (2004). Are enterovirus infections a co-factor in sudden hearing loss?. J Med Virol.

[8] Mease PJ, Ochs HD, Wedgwood RJ (1981). Successful treatment of echovirus meningoencephalitis and myositis-fasciitis with intravenous immune globulin therapy in a patient with X-linked agammaglobulinemia. N Engl J Med.

[9] Sham L, Bitnun A, Branson H, Hazrati LN, Dell SD, Yeung RSM, Johnstone J, Yeh EA (2019). Treatment of rituximab-associated chronic CNS enterovirus using IVIg and fluoxetine. Neurology.

[10] Chen H-C, Chung C-H, Wang C-H, Lin J-C, Chang W-K, Lin F-H (2017). Increased risk of sudden sensorineural hearing loss in patients with hepatitis virus infection. PLoS ONE.

